# Indonesian Translation and Cross-Cultural Adaptation of the Sensory Processing Measure-2 (SPM-2) for Preschool Children: A Study Protocol

**DOI:** 10.1155/oti/5513870

**Published:** 2025-10-27

**Authors:** Ayleen Kosasih, Putri Dirgantara, Zulfa Khoirunisah, Hermito Gidion, Muhammad Luthfi, Reza Nur Arsyi, Dini Fajariani, Herqutanto

**Affiliations:** ^1^Occupational Therapy Study Program, Vocational Higher Education Program, Universitas Indonesia, Depok, Indonesia; ^2^Department of Occupational Therapy, Graduate School of Human Health Sciences, Tokyo Metropolitan University, Tokyo, Japan; ^3^Department of Community Medicine, Faculty of Medicine, Universitas Indonesia, Jakarta, Indonesia

**Keywords:** cross-cultural, Indonesia, SPM-2, translation

## Abstract

**Introduction:**

Sensory processing is foundational for learning and behaviors. Challenges in sensory processing can impair daily functioning; therefore, sensory processing disorder is frequently linked with neurodevelopmental disorders and other clinical conditions, though it can also occur independently. However, there is a lack of validated tools in Indonesia for assessing sensory processing difficulties. The Sensory Processing Measurement second edition (SPM-2) is available in English and other languages. To ensure its accuracy for the Indonesian-speaking population, a study was conducted to translate, validate, and ensure its reliability and cultural relevance in Indonesian. This study is aimed at translating and testing the validity and reliability of the Indonesian version of the SPM-2 for preschoolers.

**Materials and Methods:**

A step-by-step approach will be conducted according to principles of good practice for the translation and cultural adaptation process for patient-reported outcome (PRO) measures from the International Society for Pharmacoeconomics and Outcomes (ISPOR). After obtaining permission from the publisher of the instrument, a sworn translator will conduct a forward translation. A native English translator will perform backward translation. Each of these translating processes will require harmonization to produce results involving a team of experts and the copyright owner. Cognitive debriefing will be conducted on parents/caregivers and teachers of 100 children ages 2–5 years. Content validity will be assessed by calculating the Item-Content Validity Index (I-CVI) and Scale-Content Validity Index (S-CVI). Moreover, reliability will be assessed using Cronbach's alpha. The result will be reviewed by the expert committee to finalize the translated document.

**Discussion:**

Translation and cross-cultural adaptation of instruments require a systematic approach to ensure their quality for research and clinical settings. Further study shall follow to test the psychometric properties of the Indonesian version of SPM-2 before it is ready for use nationwide.

## 1. Introduction

Sensory processing refers to the way the nervous system manages information received from the sensory organs to generate appropriate responses to environmental stimuli [1]. It involves detecting, regulating, and organizing input from various sensory systems: visual, auditory, tactile, olfactory, gustatory, vestibular, and proprioceptive [1, 2].

Sensory processing plays a crucial role in human function, influencing perception, behavior, and cognitive processes [3, 4]. Ayres (1972b) defined sensory processing as “the neurological process that organizes sensation from one's own body and from the environment and makes it possible to use the body effectively within the environment” [4]. It means that the brain organizes and integrates sensory information to regulate appropriate responses [5, 6]. A decreased ability to process and integrate sensory stimuli will cause difficulty in producing appropriate actions, which, in turn, may interfere with learning and behavior [4]. Consequently, sensory processing difficulties frequently interfere with functional performance across multiple domains [4]. Children and adolescents with sensory processing issues exhibit challenges in play, social participation, daily activities, education, and work [7]. Symptoms may include food selectivity, sensitivity to textures, intolerance to noise, unawareness of loud environmental sound, and poor body scheme, or may seek some sensory sensations, all of which can impair daily functioning [4].

Inefficiency in sensory processing that affects daily life is commonly referred to as sensory processing difficulties [4]. Sensory processing difficulties are frequently associated with neurodevelopmental diseases, for example, autism spectrum disorder (ASD) and attention deficit hyperactivity disorder (ADHD) [8, 9]. However, this condition may also manifest independently, without other clinical diagnoses, and is referred to as sensory processing disorder (SPD) [10]. SPD is diagnosed when an infant or young child exhibits behaviors indicating significant difficulties in processing sensory input [10]. While SPD is not formally recognized as a standalone diagnosis in major classification systems such as the DSM-5 or ICD-10, it is widely acknowledged in clinical and therapeutic settings.

There are many types of sensory processing difficulties, which are classified into two main categories: sensory modulation disorder and dyspraxia [4]. Sensory modulation disorder is a difficulty in perceiving sensory information and generating appropriate responses to the environment [4, 11]. Dyspraxia is the difficulty in planning new movement due to poor body scheme including issues with postural control [4]. To meet the diagnostic criteria, SPD symptoms must be present across multiple contexts—such as home, school, or community environments—and must significantly impact various sensory domains, including tactile, auditory, visual, vestibular, proprioceptive, olfactory, gustatory, and interoceptive processing [10].

Early identification and intervention, including occupational therapy and sensory integration (SI) strategies, are essential in helping children develop adaptive responses to sensory input, thereby enhancing their overall functional abilities and quality of life [4]. Standardized assessment tools specifically designed to evaluate sensory processing and integration are important for identifying whether sensory processing problems are present and interfering with a child's occupational performance [4]. It will provide the data about the pattern of the sensory processing difficulties of each child; this information plays a crucial role in guiding intervention planning and supports the justification for occupational therapy services utilizing an SI approach [4]. Additionally, standardized assessments can be used to measure the effectiveness of intervention programs aimed at enhancing sensory processing functions in children [4].

The Sensory Integration and Praxis Test (SIPT) is widely recognized as the gold standard for evaluating sensory processing, owing to its robust psychometric properties and extensive scope [4, 12]. This tool examines SPD through clinical observation of the performance of clients, particularly in praxis and sensory discrimination. However, this tool is expensive and may be excessive for certain situations, for example, difficulties in following instructions in the classroom [4]. As a more accessible and cost-effective alternative, the Evaluation in Ayres Sensory Integration (EASI) has been developed [13]. The EASI assesses the core Ayres Sensory Integration construct, including sensory perception, praxis, motor integration, and sensory reactivity, using open-access and low-cost materials. Preliminary studies support its construct validity and internal consistency, with international normative data collected from over 2300 children across multiple continents [13–15]. However, both EASI and SIPT do not offer a formal measurement of sensory modulation disorder [4, 13]. Thus, additional evaluation using standardized caregiver questionnaires, such as the sensory profile (SP) [16] and Sensory Processing Measure (SPM) [17], is required to complete the assessment [4]. These assessments, reported by parents and teachers, evaluate sensory processing and modulation, body awareness, and praxis, along with the functional consequences of challenges in each area [4]. When sensory modulation disorder is suspected, SP or SPM is recommended as the assessment tool [4]. The preschool Sensory Processing Measure-2 (SPM-2) is designed for children aged 2–5 years. This assessment demonstrates strong test–retest reliability, with a reliability correlation coefficient of 0.82–0.96 (SEM = 1.73–3.80) and 0.75–0.95 (SEM = 2.39–4.45) for home and school form, respectively [9]. This tool also scored high for other measures of reliability as well as multiple aspects of validity [9]. Additionally, the SPM-2 specifically identifies challenges in participation at home, school, and in the community that are believed to stem from difficulties in sensory processing [4, 9]. Measurement across different environments provides comprehensive information on the sensory processing of an individual [18].

While these assessment tools demonstrate high validity and reliability, they are based on the caregiver's perception of the client's performance which may compromise their objectivity. Moreover, their normative data primarily reflect North American children [4, 12]. This geographical and cultural specificity may yield different outcomes when these tools are applied in diverse settings, potentially impacting their validity in other cultural and environmental contexts [12]. Consequently, clinicians should exercise caution when using these assessments with children outside of North America, ensuring they incorporate their professional judgment and direct observations into their decision-making processes.

Currently, Indonesia lacks an official sensory screening tool, complicating assessments. Although Yudhiatmoko [19] previously translated and tested the Short Sensory Profile for Indonesian children, there remains a deficiency in culturally validated tools. The SPM-2, a tool used internationally from infancy to adulthood, has been translated for use in countries like Saudi Arabia [20] and Sweden [21]. This study is aimed at translating and culturally validating the SPM-2 for Indonesian children aged 2–5 years to provide an appropriate tool for assessing sensory processing.

## 2. Methods

The study will be carried out in the Greater Jakarta Metropolitan area (Jabodetabek). As SPM-2 comprises two forms, home and school form, these will be filled out by parents/caregivers and teachers. Ethical approval has been obtained from the RSUI Ethics Committee (No. S-129/KETLIT/RSUI/VIII/2024). Informed consent will be obtained from the participants prior to the study.

The inclusion criteria for the study are as follows: children aged 2–5 years who reside in the Greater Jakarta Metropolitan area. Parents or caregivers must have at least a middle school education. Furthermore, the teachers must be preschool teachers. The exclusion criteria for both parents and teachers are those who do not understand the Indonesian language or have medical/mental conditions which complicate the cognitive debriefing process.

**Figure 1:**
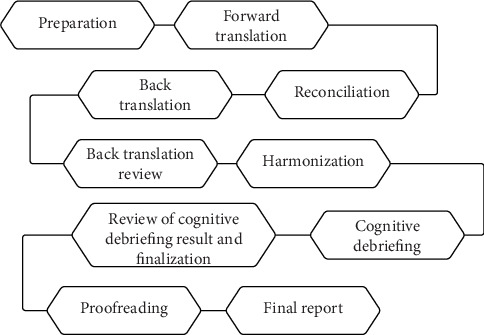
Flowchart of the ISPOR-guided translation process. The 10-step approach ensures that the process is done systematically and reviewed thoroughly.

**Figure 2:**
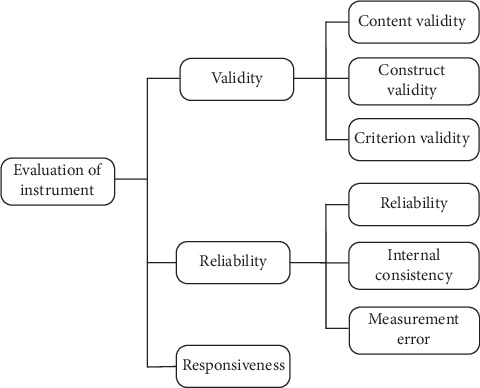
Evaluation framework for outcome measurement instruments based on COSMIN recommendations. A comprehensive assessment should examine the validity, reliability, and responsiveness of an instrument to ensure its measurement accuracy and consistency.

The translation and adaptation procedure follows International Society for Pharmacoeconomics and Outcomes (ISPOR) principles of good practice for the translation and cultural adaptation process for patient-reported outcome (PRO) measures [22] and COnsensus-based Standards for the selection of health Measurement INstruments (COSMIN) [23] with slight modification. The ISPOR is a global organization focused on advancing health economics and outcome research to inform healthcare policy and practice [24]. As shown in Figure 1, the ISPOR guideline recommends a comprehensive approach beginning with dual independent forward translations, reconciliation by a subject matter expert, and subsequent back translation to verify semantic equivalence. This is followed by cognitive debriefing with the target population to ensure cultural and contextual appropriateness and a final review to consolidate all adaptations before proofreading and documentation [22]. Meanwhile, COSMIN is an initiative aimed at enhancing the quality of health measurement instruments. It provides methodological standards for validating health measurement tools, emphasizing rigorous testing of validity and reliability, and reporting in diverse settings [25]. The COSMIN guidelines emphasize ensuring methodological rigor through detailed testing of the content and construct validity, reliability, and reporting processes (Figure 2). Content validity is deemed the most crucial aspect, serving as the foundation to ensure the instrument's items are appropriate and cover all facets of the construct being measured [25]. This is followed by assessments of the internal structure, which includes structural validity to ascertain the dimensionality of the construct, internal consistency to ensure item coherence within the instrument, and cross-cultural validity to verify the instrument's applicability across different cultural contexts. Reliability is also a key focus, involving repeated measures to confirm stability and consistency of the instrument over time and across various raters. Following this, criterion validity evaluates the instrument's efficacy by comparing it with a gold standard, where possible. Hypothesis testing for construct validity is conducted to verify theoretical predictions about the relationships that should exist with other measures or different groups. Lastly, the responsiveness of the instrument is tested to ensure that it can detect change over time in the construct of interest [25]. These steps involve not only translation and adaptation to the new cultural context but also systematic validation to confirm that the tool accurately measures the intended constructs and maintains consistency across different settings and times [23]. Both guidelines underscore the necessity of involving both linguistic and subject matter experts throughout the process to achieve a robust and reliable adapted assessment tool.

### 2.1. Translation and Cross-Cultural Adaptation Process

#### 2.1.1. Step 1: Preparation

In the preparation step, the research team contacts Western Psychological Services (WPS) as the publisher of SPM-2 for permission to undergo the translation and cross-cultural adaptation process to the Indonesian language. Upon the approval of the publisher, an expert committee will be formed, consisting of six occupational therapists, four educators of preschool children, and four parents of children between 2 and 5 years of age.

Occupational therapists will provide their expertise in the practical application of the assessment tool within therapeutic settings. Their professional insights ensure that the translation accurately reflects the terminology and constructs essential to occupational therapy, preserving the tool's evaluative integrity and functionality in a clinical context.

Preschool educators will offer critical input on the appropriateness of language and concepts for young children and their caregivers. Their educational expertise facilitates an adaptation that aligns with developmental standards and educational clarity, ensuring that the tool is both comprehensible and accessible for preschool-aged children and their families in Indonesia.

Parents of young children will contribute their real-world perspectives, highlighting the practical implications of the translation in everyday contexts. Their input helps to confirm that the tool's language and scenarios are relatable, culturally sensitive, and aligned with Indonesian familial structures, thereby enhancing the authenticity and acceptability of the assessment within the intended demographic.

#### 2.1.2. Step 2: Forward Translation

Forward translation will be performed by a single sworn professional translator into Indonesian. The translator is of Indonesian nationality. Using a single sworn professional translator ensures that the language transfer is accurate, consistent, and legally validated. Although ISPOR recommends two translators with experience in PRO measures, a sworn translator offers certified proficiency in language skills and familiarity with formal translation standards. This approach maintains a high-quality translation and reduces potential inconsistencies that may arise from using two separate translators.

#### 2.1.3. Step 3: Reconciliation

Following the translation process, the expert committee will hold a focus group discussion (FGD) to assess the semantic and conceptual equivalence of the Indonesian version. This panel structure is chosen to ensure that the translation not only preserves the assessment tool's intended meaning but also resonates with the daily realities and cultural nuances of the target population.

During the discussion, members can suggest changes of phrases or sentences to be adaptable in the cross-cultural translation process. All inputs will be recorded and discussed for acceptance or rejection. We will review the original SPM-2 and evaluate how well it aligns with Indonesia's cultural norms and environmental factors. This involves a detailed examination of specific items to identify potential differences in interpretation. For instance, we are looking at how children interact with their environment and at the role of cultural expectations around group behavior, obedience, and noise tolerance. An example of a potential difference in interpretation is an item like “Child is uncomfortable with loud noises in the environment,” which may require careful adaptation, as the perception of “loud noise” can differ significantly between rural and urban settings in Indonesia.

#### 2.1.4. Step 4: Back Translation

After finishing the FGD process, the first draft of SPM-2 has been established. Then, the second translator will conduct a back translation of the first draft of the SPM-2 Indonesian version. The translator is a native English speaker blinded to the original version of SPM-2. This translator is intentionally blinded to the original English version of the SPM-2, a measure designed to avoid bias and ensure that the back translation reflects only the content of the translated draft. This step enhances the translation's fidelity, allowing for a comparison with the original version and identification of any discrepancies that may require further refinement.

#### 2.1.5. Step 5: Back Translation Review

This back-translated version will be sent to WPS for review of the conceptual equivalence with the original version. This review focuses on assessing the conceptual equivalence between the back-translated version and the original SPM-2. By comparing these versions, WPS can identify any discrepancies or misunderstandings in translation, ensuring that the adapted tool maintains the intended meaning and relevance of the original instrument. This step is crucial for validating the accuracy and cultural appropriateness of the translated assessment.

#### 2.1.6. Step 6: Harmonization

During this step, queries from WPS will be clarified by the research team, in writing or, if necessary, in a meeting. Clarifications are provided in writing to maintain a clear record, and if required, a meeting will be conducted to ensure mutual understanding. This collaborative step ensures that any ambiguities or cultural nuances in the translation are fully resolved, aligning both teams on the final content and enhancing the consistency and reliability of the adapted SPM-2 version.

#### 2.1.7. Step 7: Cognitive Debriefing

The next step is cognitive debriefing or a pilot study to test the validity and reliability of the Indonesian version of the instrument. Parents/caregivers and teachers of 100 children aged 2–5 years will be selected by purposive sampling. This size balances the accuracy of the reliability measures with the practical constraints of research, providing a sufficient number of data points to minimize the impact of outliers and increase the generalizability of the findings [19–21]. We will enlist kindergartens and early childhood education centers (PAUD), preferably inclusive schools, in the study area. We will select the schools as the study site based on logistical consideration and their cooperativeness. Upon obtaining permission from the selected schools, we will invite prospective participants (parents/caregivers and teachers) to attend a school meeting. During this meeting, we will provide detailed explanations of the study's objectives in the form of a seminar, followed by a discussion session. The topics covered are the significance and goals of the SPM-2 preschool research, the study procedures, benefits for participants, inclusion and exclusion criteria, and the research process and schedule. We will obtain informed consent from the prospective participants and collect their demographic information. All consenting participants will be asked to complete the SPM-2 forms, with an open invitation to provide any additional comments or feedback regarding the SPM-2 process. The research team will be available during the filling out of the forms to provide clarification or further information when needed.

#### 2.1.8. Step 8: Review of Cognitive Debriefing Results and Finalization

After the required number of participants is met, we will analyze the content validity and reliability of the instrument. We will conduct content validity index (CVI) to evaluate the relevance and representativeness of the items in the instrument. Each expert committee member will independently rate the relevance of each item on a 4-point scale, ranging from 1 (*not relevant*) to 4 (*highly relevant*). We will calculate Item-Content Validity Index (I-CVI) for each item, with values of 0.78 or higher considered acceptable. Additionally, we will calculate the Scale-Content Validity Index (S-CVI) using both the universal agreement (S-CVI/UA) and average CVI (S-CVI/Ave) methods. Moreover, to assess the internal consistency of SPM-2, we will calculate Cronbach's alpha; a value above 0.70 is generally considered acceptable, with higher values suggesting greater internal consistency.

#### 2.1.9. Step 9: Proofreading

After completing the procedure, we will conduct a rigorous proofreading process to ensure the final version's accuracy, clarity, and cultural relevance. This stage involves a thorough review by bilingual experts who are not involved in the initial translation process to provide an independent assessment of the translated content. The proofreading team meticulously examines the instrument for any linguistic inconsistencies, ambiguities, or cultural discrepancies that could affect its validity. Feedback from this review will be used to make minor adjustments to the language, ensuring that the instrument retains its original meaning while being fully comprehensible and culturally appropriate for the target population.

#### 2.1.10. Step 10: Final Report

After proofreading, we will compile a final report, documenting the entire translation and adaptation process. This report includes details on each stage, from initial translation and back-translation to expert committee review, pilot testing, and final revisions. The report serves as a comprehensive record, demonstrating the methodological rigor and thoroughness applied to ensure the instrument's suitability for use in the new cultural context.

## 3. Discussion

Sensory processing is crucial for the normal development of human beings. Thus, assessment of this aspect is important in dealing with developmental delay and other clinical cases involving sensory processing difficulties. Currently, the widely used instruments for such purpose are available in English, whereas such measurement is local bias, whether it is language or cultural. Thus, there is an unquestionable need to provide the tool in Indonesian. This underscores the significant imperative for localized versions of these tools, particularly in Indonesia, where cultural and environmental factors substantially impact the expression of sensory processing issues. We perceived this need and formulated a clearly defined and systematic protocol to translate SPM-2, one of the widely used instruments to assess sensory processing for children. This protocol will provide a guideline, particularly for therapists and researchers in the field of pediatrics, aiming to enrich the availability of such instruments in the Indonesian language.

The translation process involves a thorough review of the original SPM-2 and an assessment of its alignment with Indonesia's cultural norms and environmental factors. This involves a detailed examination of specific items to identify potential differences in interpretation. For instance, we are looking at how children interact with their environment and at the role of cultural expectations around group behavior, obedience, and noise tolerance.

The next step involves translating these items in a culturally appropriate and understandable manner for both practitioners and families. This process includes several phases, such as back-translation, cognitive debriefing, and testing the translated version for reliability and validity. A key challenge will be ensuring that culturally specific behaviors and expressions are accurately captured in the Indonesian version to reflect the diverse experiences of children across different regions.

The limitations of this study are mainly related to cultural variability, sample size, and the scope of the reliability testing. While the translation is aimed at adapting the SPM-2 for the Indonesian context, the cultural diversity within Indonesia itself may impact the applicability and generalizability across different regions, where norms and sensory processing perceptions can vary significantly. The sample used in the pilot study, comprising 100 children aged 2–5 years from Greater Jakarta Metropolitan, may not fully represent the broader Indonesian population, thus restricting the generalizability of the findings to other areas with diverse cultural and socioeconomic backgrounds.

Additionally, while the CVI and Cronbach's alpha are used to assess the content validity and internal consistency of the tools, it is important to note that these measures may not fully encompass all aspects of the tools' psychometric properties. Further testing, including test–retest reliability and construct validity, is necessary. The process of translating and culturally adapting the tools also presents challenges, as certain culturally specific behaviors and perceptions may not be accurately captured without losing some of the original instrument's meaning.

Our goal is to maintain the SPM-2's validity and reliability while adapting it to Indonesia's unique cultural landscape. This effort is crucial for ensuring that the SPM-2 can be effectively used in both educational and clinical settings, providing more accurate assessments and better support for children with sensory processing issues in Indonesia.

## 4. Conclusion

The translation and cultural adaptation of the SPM-2 into Indonesian is a significant step toward providing reliable and valid tools for assessing sensory processing issues in children aged 2–5 years in Indonesia. This study protocol outlines a systematic process that adheres to established principles for translation and cross-cultural adaptation, ensuring that the adapted version is culturally relevant and linguistically appropriate. Further studies are necessary to confirm its psychometric properties across diverse populations and settings within Indonesia, ultimately supporting its nationwide implementation for early identification and intervention in sensory processing difficulties.

## Data Availability

Data sharing is not applicable to this article as no new data were created or analyzed in this study.
